# Comparison of the prognostic value of selected markers of the systemic inflammatory response in patients with colorectal cancer

**DOI:** 10.1038/sj.bjc.6604027

**Published:** 2007-10-09

**Authors:** E F Leitch, M Chakrabarti, J E M Crozier, R F McKee, J H Anderson, P G Horgan, D C McMillan

**Affiliations:** 1 University Department of Surgery, Royal Infirmary, Glasgow G31 2ER, UK

**Keywords:** colorectal cancer, TNM stage, C-reactive protein, albumin, white cells, survival

## Abstract

There is increasing evidence that the presence of a systemic inflammatory response plays an important role in predicting survival in patients with colorectal cancer. However, it is not clear what components of the systemic inflammatory response best predict survival. The aim of the present study was to compare the prognostic value of an inflammation-based prognostic score (modified Glasgow Prognostic Score (Mgps) 0=C-reactive protein <10 mg l^−1^, 1=C-reactive protein >10 mg l^−1^, and 2=C-reactive protein >10 mg l^−1^ and albumin<35 g l^−1^) with that of components of the white cell count (neutrophils, lymphocytes, monocytes and platelets using standard thresholds) in patients with colorectal cancer. Two patient groups were studied: 149 patients who underwent potentially curative resection for colorectal cancer and 84 patients who had synchronous unresectable liver metastases. In those patients who underwent potentially curative resection the minimum follow-up was 36 months and 20 patients died of their cancer. On multivariate survival analysis only TNM stage (HR 3.75, 95% CI 1.54–9.17, *P*=0.004), monocyte count (HR 3.79, 95% CI 1.29–11.12, *P*=0.015) and mGPS (HR 2.21, 95% CI 1.11–4.41, *P*=0.024) were independently associated with cancer-specific survival. In patients with synchronous unresectable liver metastases the minimum follow-up was 6 months and 71 patients died of their cancer. On multivariate survival analysis only single liver metastasis >5 cm (HR 1.78, 95% CI 0.99–3.21, *P*=0.054), extra-hepatic disease (HR 2.09, 95% CI 1.05–4.17, *P*=0.036), chemotherapy treatment (HR 2.40, 95% CI 1.82–3.17, *P*<0.001) and mGPS (HR 1.44, 95% CI 1.01–2.04, *P*=0.043) were independently associated with cancer-specific survival. In summary, markers of the systemic inflammatory response are associated with poor outcome in patients with either primary operable or synchronous unresectable colorectal cancer. An acute-phase protein-based prognostic score, the mGPS, appears to be a superior predictor of survival compared with the cellular components of the systemic inflammatory response.

Colorectal cancer remains the second commonest cause of cancer death in Western Europe and North America. Each year in the United Kingdom, there are approximately 35 000 new cases and 16 000 deaths attributable to the disease (Cancerstats, 2004, www.cancerresearchuk.org). Approximately one-fifth of patients will have colorectal liver metastases. Overall survival is poor; even in those patients who undergo potentially curative resection, more than one-third die within 5 years (McArdle and Hole, 2002).

It is increasingly recognised that variations in outcome in cancer patients are not solely determined by the characteristics of the tumour but also by the host-response factors (MacDonald, 2007). It is now accepted that the host systemic inflammatory response can be assessed by examining the changes in the circulating concentrations of acute-phase proteins, such as elevated concentration of C-reactive protein and low concentrations of albumin (Gabay and Kushner, 1999; McMillan *et al*, 2001). It is of interest that either singly or combined these factors have been shown to be stage- and performance status-independent prognostic factors in patients with a variety of inoperable cancers (Forrest *et al*, 2003, 2004; Al Murri *et al*, 2006; Crumley *et al*, 2006; Glen *et al*, 2006; Ramsey *et al*, 2007). Similarly, singly these factors have been shown to be tumour stage-independent prognostic factors, prior to surgery, in patients with a variety of primary operable cancers including colorectal cancer (Heys *et al*, 1998; Longo *et al*, 1998; Nozoe *et al*, 1998; Longo *et al*, 2000; Nielsen *et al*, 2000; McMillan *et al*, 2003). Furthermore, Wong *et al* (2007) showed that an elevated C-reactive protein concentration was an independent predictor of survival in patients undergoing potentially curative surgery for colorectal liver metastases. Recently, the combination of C-reactive protein and albumin, known as the Glasgow Prognostic Score (GPS) has been evaluated pre-operatively in patients undergoing potentially curative surgery for colorectal cancer (McMillan *et al*, 2007).

However, acute-phase proteins are just one aspect of the systemic inflammatory response (Gabay and Kushner, 1999). There are cellular components of the systemic inflammatory response such as neutrophils, lymphocytes, monocytes and platelets that have been reported to have prognostic value in patients with a variety of common solid tumours (Riesco, 1970; Bruckner *et al*, 1982; Vigano *et al*, 2000; Maltoni *et al*, 2005; Hauser *et al*, 2006). Therefore, it may be that the components of the whole-blood differential white cell count either singly or in combination may have prognostic value in patients with either primary operable or metastatic colorectal cancer. Indeed, Walsh *et al* (2005) reported that the neutrophil/lymphocyte ratio had prognostic value in patients undergoing surgery for colorectal cancer. However, it is known whether such white cell measures offer prognostic value that is independent of tumour stage or superior to the combination of C-reactive protein and albumin (GPS).

Therefore, the aim of the present study was to examine the relationship between selected markers of the systemic inflammatory response, treatment and survival in patients with primary operable or synchronous unresectable colorectal cancer.

## PATIENTS AND METHODS

### Patients

Patients with histologically proven colorectal cancer who, on the basis of laparotomy findings and/or preoperative abdominal-computed tomography, were considered to have undergone a potentially curative resection or had synchronous unresectable liver metastases and had routine laboratory measurement of white cell, neutrophil, lymphocyte, monocyte and platelet counts, albumin and C-reactive protein, between February 1998 and May 2006 at Glasgow Royal Infirmary were included in the study. The tumours were staged according to the TNM criteria (AJCC, 2002). Patients who had clinical evidence of infection or other inflammatory condition were excluded from the study.

The extent of deprivation was defined using the Carstairs deprivation index (Carstairs and Morris, 1991). This is an area-based measure derived from the 1991 census, using the postcode of residence at diagnosis, which divides the score into a seven-point index. For illustrative purposes, the results are presented by amalgamating the seven categories into three groups: affluent (categories 1 and 2), intermediate (categories 3–5) and deprived (categories 6 and 7). The Carstairs deprivation index has been extensively utilised in cancer patients and is particularly appropriate for use in the central belt of Scotland (Hole and McArdle, 2002).

Patients with synchronous colorectal liver metastases either had their primary colorectal cancer excised, stented, or by-passed due to obstructive symptoms or chemo/radiotherapy.

The study was approved by the Research Ethics Committee, Royal Infirmary, Glasgow.

### Methods

The coefficient of variation for the routine laboratory measurements of absolute white cell, neutrophil, lymphocyte, monocyte and platelet counts, albumin and C-reactive protein, over the range of measurement, was less than 10% as established by routine quality control procedures. The limit of detection of the assay was a C-reactive protein concentration lower than 6 mg l^−1^.

The GPS was constructed as previously described (Forrest *et al*, 2003). Briefly, patients with both an elevated C-reactive protein (>10 mg l^−1^) and hypoalbuminaemia (<35 g l^−1^) were allocated a score of 2. Patients in whom only one of these biochemical abnormalities was present were allocated a score of 1. Patients in whom neither of these abnormalities was present were allocated a score of 0.

Recently, however, this has been modified based on evidence that hypoalbuminaemia, in patients without an elevated C-reactive protein concentration, had no significant association with cancer-specific survival. Therefore, patients with an elevated C-reactive protein were assigned a modified GPS score (mGPS) of 1 or 2 depending on the absence or presence of hypoalbuminaemia (McMillan *et al*, 2007).

### Statistics

Grouping of the variables white cell, neutrophil, lymphocyte, monocyte and platelet counts was carried out using standard thresholds (Riesco, 1970; Bruckner *et al*, 1982; Vigano *et al*, 2000; Maltoni *et al*, 2005; Hauser *et al*, 2006). Survival analysis of the group variables was performed using the Cox proportional hazard model. Deaths up to the end of April 2007 were included in the analysis. Multivariate survival analysis, including all significant covariates (*P*<0.05 to account for multiple comparisons) was performed using a stepwise backward procedure to derive a final model of the variables that had a significant independent relationship with survival. To remove a variable from the model, the corresponding *P*-value had to be greater than 0.05. The relationships between the mGPS and other variables were analysed using the Mantel–Haenszel (X^2^) test for trend as appropriate. Analysis was performed using SPSS software (SPSS Inc., Chicago, IL, USA).

## RESULTS

Two patient groups were studied: 149 patients who underwent potentially curative resection for colorectal cancer and 84 patients who had synchronous unresectable liver metastases (Table 1). Overall, the majority of patients were aged 65 years or more, were male, were deprived, had colonic tumours and had TNM stage I/II tumours. The majority of patients had white cell, neutrophil, lymphocyte, monocyte and platelet counts and albumin concentrations in the normal range. In contrast, C-reactive protein and thus the mGPS were elevated in the majority of patients.

Patients with synchronous unresectable liver metastases had higher white cell, neutrophil, lymphocyte and monocyte counts (all *P*<0.01) and C-reactive and mGPS (both *P*<0.001) compared with primary operable disease (Table 1). On follow-up more patients with synchronous unresectable liver metastases died of their cancer (*P*<0.001).

The relationship between clinicopathological characteristics, systemic inflammatory response and survival in patients with primary operable cancer is shown in Table 2. The minimum follow-up was 36 months, maximum 73 months; the median follow-up of the survivors was 48 months. During the follow-up period 45 patients died, 20 patients of their cancer and 25 of intercurrent disease. On univariate survival analysis, age (*P*<0.01), TNM stage (*P*<0.05), white cell (*P*⩽0.001), neutrophil (*P*<0.01), monocyte (*P*<0.01), and platelet (*P*<0.05) counts and mGPS (*P*⩽0.001) were significantly associated with overall survival (Table 2). On multivariate survival analysis of these significant variables only age (HR 1.74, 95% CI 1.19–2.56, *P*=0.005), TNM stage (HR 2.28, 95% CI 1.36–3.82, *P*=0.002), monocyte count (HR 3.11, 95% CI 1.42–6.82, *P*=0.005) and mGPS (HR 2.08, 95% CI 1.32–3.28, *P*=0.002) were independently associated with overall survival.

On univariate survival analysis, TNM stage (*P*<0.05), white cell (*P*<0.01), neutrophil (*P*<0.01), monocyte (*P*⩽0.01), and platelet (*P*<0.05) counts and mGPS (*P*<0.05) were significantly associated with poorer cancer-specific survival (Table 2). On multivariate survival analysis of these significant variables, only TNM stage (HR 3.75, 95% CI 1.54–9.17, *P*=0.004), monocyte count (HR 3.79, 95% CI 1.29–11.12, *P*=0.015) and mGPS (HR 2.21, 95% CI 1.11–4.41, *P*=0.024) were independently associated with cancer-specific survival. When mGPS was excluded from the model, on multivariate analysis only TNM stage (HR 2.49, 95% CI 1.11–5.61, *P*=0.0274) and white cell count (HR 2.22, 95% CI 1.28–3.84, *P*=0.004) were independently associated with cancer-specific survival.

The relationship between clinicopathological characteristics, systemic inflammatory response and survival in patients with unresectable colorectal liver metastases is shown in Table 3. The minimum follow-up was 6 months, maximum 73 months; the median follow-up of the survivors was 12 months. A total of 71 (85%) patients died of their disease during the follow-up period. On univariate analysis, age (*P*<0.05), any single liver metastasis >5 cm (*P*<0.01), extra-hepatic disease (*P*<0.05), chemotherapy (*P*<0.0001) and mGPS (*P*⩽0.001) were significantly associated with poorer cancer-specific survival (Table 3). On multivariate analysis of these significant variables, including treatment, only single liver metastasis >5 cm (HR 1.78, 95% CI 0.99–3.21, *P*=0.054), extra-hepatic disease (HR 2.09, 95% CI 1.05–4.17, *P*=0.036), chemotherapy treatment (HR 2.40, 95% CI 1.82–3.17, *P*<0.001) and mGPS (HR 1.44, 95% CI 1.01–2.04, *P*=0.043) were independently associated with poorer cancer-specific survival. When mGPS was excluded from the model, on multivariate analysis only single liver metastasis >5 cm (HR 1.97, 95% CI 1.12–3.47, *P*=0.018), extra-hepatic disease (HR 2.02, 95% CI 1.02–4.01, *P*=0.043) and chemotherapy treatment (HR 2.29, 95% CI 1.76–2.98, *P*<0.001) were independently associated with cancer-specific survival.

The relationship between the mGPS and clinicopathological characteristics in all colorectal cancer patients is shown in Table 4. An increase in the mGPS was associated with an increase in TNM stage (*P*<0.001), higher white cell (*P*<0.001), neutrophil (*P*<0.001), monocyte (*P*<0.01) and platelet (*P*<0.001) counts and also the neutrophil lymphocyte ratio (*P*<0.001). An increase in the mGPS was associated with a lower lymphocyte count (*P*<0.01).

**Figure 1:**
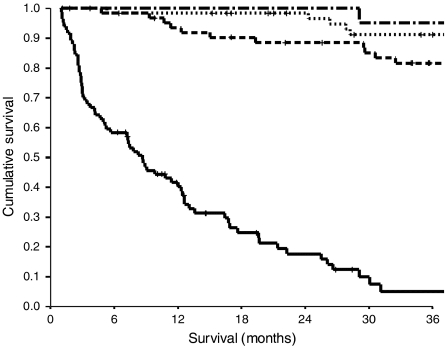
The relationship between TNM stage (I, II, III and IV from top to bottom) and cancer-specific survival in patients with colorectal cancer.

**Figure 2:**
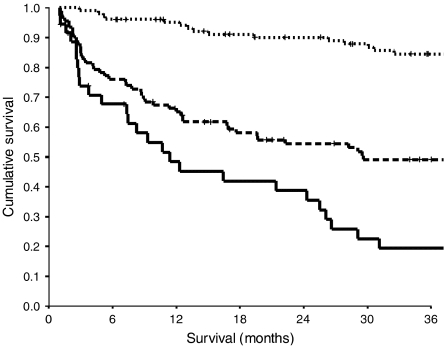
The relationship between mGPS (0, 1 and 2 from top to bottom) and cancer-specific survival in patients with colorectal cancer.

The relationship between TNM stage, the mGPS and cancer-specific survival is shown in Figures 1 and 2 respectively.

## DISCUSSION

In the present study a number of cellular components of the systemic inflammatory response were associated with cancer-specific survival in patients with colorectal cancer and therefore confirm previous work in other tumour types. However, compared with a score (mGPS) based on the acute-phase proteins, C-reactive protein and albumin, these cellular components of the white cell count, did not consistently have independent prognostic value. Therefore, it would appear that the mGPS is superior to these cellular components, in particular the neutrophil/lymphocyte ratio, in predicting survival at different tumour stages in patients with colorectal cancer.

The results of the present study may also offer insight into the nature of the relationship between the systemic inflammatory response and survival in patients with primary operable and metastatic unresectable colorectal cancer. A plausible explanation is that an elevated mGPS may reflect compromised acquired immunity as it was associated with an increase in lymphopenia. An alternative explanation is that an elevated mGPS may reflect altered innate immunity as it was also associated with increased numbers neutrophils and monocytes. However, given that higher neutrophil and monocyte counts were more strongly associated with poorer cancer-specific survival this may suggest that of activation of the innate, rather than downregulation of the acquired immune system, is the most important factor in determining poor outcome in patients with colorectal cancer. It may be that downregulation of these innate cell types should targeted in the treatment of patients with colorectal cancer.

With respect to metastatic unresectable disease there is now considerable evidence that chronic activation of the systemic inflammatory response is associated with an increase in weight loss, in particular the loss of lean tissue, with the consequent increase in fatigue, decreased performance status and survival (Kotler and Cachexia, 2000; MacDonald, 2007).

Irrespective of the mechanisms involved, we believe that the presence or absence of a systemic inflammatory response as measured by the mGPS should be evaluated, prior to treatment, in patients with colorectal cancer and should be used in the risk stratification of such patients. In summary, markers of the systemic inflammatory response are associated with poor outcome in patients with either primary operable or synchronous unresectable colorectal cancer. An acute-phase protein-based prognostic score, the mGPS, appears to be a superior predictor of survival compared with cellular components of the systemic inflammatory response.

## Figures and Tables

**Table 1:** The relationship between clinicopathological characteristics, the systemic inflammatory response and survival in patients with colorectal cancer

	**Primary operable (*n*=149)**	**Synchronous unresectable (*n*=84)**	***P*-value**
Age (<65/65–74/>75 years)	48/52/49	34/27/23	0.215
Sex (male/female)	81/68	48/36	0.683
Deprivation (1–2/3–5/6–7)^a^	5/66/78	7/33/44	0.981
Tumour site (colon/rectum)	83/66	52/32	0.358
TNM Stage (I/II/III/IV)	22/62/65/0	0/0/0/84	<0.001

White cell count (<8.5/8.5–11/>11 × 10^9^ l^−1^)	82/40/17	30/27/27	<0.001
Neutrophil count (<7.5/>7.5 × 10^9^ l^−1^)	129/20	49/35	<0.001
Lymphocyte count (>3.0/1.0–3.0/<1.0 × 10^9^ l^−1^)	7/131/11	2/66/16	0.008
Neutrophil/lymphocyte ratio (<5/>5)	124/25	48/36	<0.001
Monocyte count (<0.9/>0.9 × 10^9^ l^−1^)	132/17	61/23	0.002
Platelet count (<400/>400 × 10^3^ l^−1^)	117/32	55/27	0.057

C-reactive protein (<10/>10 mg l^−1^)	88/61	17/64	<0.001
Albumin (>35/<35 g l^−1^)	135/14	55/29	<0.001
MGPS (0/1/2)	88/48/13	17/44/23	<0.001

Alive	104	13	
*Dead*			
Cancer	20	71	
Non-cancer	25	0	<0.001

TNM=tumour, node, metastases; mGPS=modified Glasgow Prognostic Score.

a Individual deprivation categories were used in the statistical analysis.

**Table 2:** The relationship between clinicopathological characteristics, the systemic inflammatory response and survival in patients with primary operable colorectal cancer; univariate analysis

		**Overall survival**		**Cancer-specific survival**	
	**Patients (*n*=149)**	**Hazard ratio (95% CI)**	***P*-value**	**Hazard ratio (95% CI)**	***P*-value**
Age (<65/65–74/⩾75 years)	48/52/49	1.65 (1.13–2.39)	0.009	1.48 (0.85–2.57)	0.166
Sex (male/female)	81/68	0.76 (0.42–1.38)	0.371	1.15 (0.48–2.75)	0.762
Deprivation (1-2/3–5/6–7)^a^	5/66/78	0.98 (0.82–1.16)	0.799	0.87 (0.68–1.13)	0.298
Tumour site (colon/rectum)	83/66	0.96 (0.54–1.74)	0.903	0.80 (0.33–1.95)	0.621
TNM Stage (I/II/III)	22/62/65	1.79 (1.13–2.85)	0.014	2.63 (1.19–5.80)	0.016
Neo-adjuvant therapy (yes/no)	11/138	0.55 (0.13–2.25)	0.402	1.30 (0.30–5.62)	0.722
Adjuvant therapy (yes/no)	52/97	0.89 (0.48–1.66)	0.718	1.79 (0.74–4.29)	0.195

White cell count (<8.5/8.5–11/>11 × 10^9^ l^−1^)	92/40/17	1.92 (1.32–2.81)	0.001	2.40 (1.38–4.16)	0.002
Neutrophil count (<7.5/⩾7.5 × 10^9^ l^−1^)	129/20	2.90 (1.46–5.73)	0.002	3.77 (1.44–9.82)	0.007
Lymphocyte count (>3.0/1.0–3.0/<1.0 × 10^9^ l^−1^)	7/131/11	0.72 (0.29–1.80)	0.484	0.30 (0.09–1.01)	0.053
Monocyte count (⩽0.9/>0.9 × 10^9^ l^−1^)	132/17	2.86 (1.38–5.95)	0.005	3.78 (1.37–10.40)	0.010
Platelet count (<400/⩾400 × 10^3^ l^−1^)	117/32	2.10 (1.12–3.95)	0.022	2.53 (1.01–6.35)	0.048

Neutrophil/lymphocyte ratio (<5/⩾5)	124/25	1.68 (0.83–3.39)	0.150	1.49 (0.50–4.45)	0.479
mGPS (0/1/2)	88/48/13	2.07 (1.35–3.16)	0.001	2.01 (1.06–3.80)	0.032

TNM=tumour, node, metastases; mGPS=modified Glasgow Prognostic Score; CI=confidence interval.

a Individual deprivation categories were used in the statistical analysis.

**Table 3:** The relationship between clinicopathological characteristics, the systemic inflammatory response and cancer-specific survival in patients with synchronous unresectable colorectal liver metastases; univariate analysis

	**Patients (*n*=84)**	**Hazard ratio (95% CI)**	***P*-value**
Age (<65/65–74/⩾75 years)	34/27/23	1.47 (1.08–2.00)	0.0139
Sex (male/female)	48/36	1.13 (0.70–1.82)	0.6176
Deprivation (1–2/3–5/6–7)^a^	7/33/44	0.98 (0.85–1.12)	0.7336
Tumour site (colon/rectum)	52/32	1.14 (0.70–1.85)	0.6048
Emergency presentation (no/yes)	75/9	1.56 (0.74–3.27)	0.2423
No. of liver metastases (1/>1)	4/80	2.05 (0.64–6.57)	0.2270
Any single liver metastasis (⩽5/>5 cm)	36/38	2.19 (1.30–3.67)	0.0030
Extra-hepatic disease (no/yes)	64/20	2.06 (1.16–3.67)	0.0140
Therapy: chemotherapy+primary resected or stent/chemotherapy alone/primary resected or stent alone/no active treatment	28/20/23/13	2.16 (1.71–2.73)	<0.0001
White cell count (<8.5/8.5–11/>11 × 10^9^ l^−1^)	30/27/27	1.31 (0.99–1.75)	0.0631
Neutrophil count (<7.5/⩾7.5 × 10^9^ l^−1^)	49/35	1.57 (0.98–2.53)	0.0627
Lymphocyte count (>3.0/1.0–3.0/<1.0 × 10^9^ l^−1^)	2/66/16	1.65 (0.93–2.93)	0.0864
Monocyte count (⩽0.9/>0.9 × 10^9^ l^−1^)	61/23	1.32 (0.77–2.25)	0.3122
Platelet count (<400/⩾400 × 10^3^ l^−1^)	55/27	1.36 (0.82–2.25)	0.2338
Neutrophil/lymphocyte ratio (<5/⩾5)	48/36	1.35 (0.84–2.16)	0.2200
mGPS (0/1/2)	17/44/23	1.46 (1.05–2.03)	0.0243

mGPS=modified Glasgow Prognostic Score; CI=confidence interval.

a Individual deprivation categories were used in the statistical analysis.

**Table 4:** The relationship between the mGPS and clinicopathological characteristics in patients with colorectal cancer (*n*=233)

	**mGPS 0 (*n*=105)**	**mGPS 1 (*n*=92)**	**mGPS 2 (*n*=36)**	***P*-value**
Age (<65/65–74/>75 years)	37/38/30	34/30/28	11/11/14	0.431
Sex (male/female)	62/43	47/45	20/16	0.487
Deprivation (1–2/3–5/6–7)^a^	6/45/54	4/41/47	2/13/21	0.584
Tumour site (colon/rectum)	54/51	59/33	22/14	0.142
TNM Stage (I/II/III/IV)	15/30/43/17	6/25/17/44	1/7/5/23	<0.001
White cell count (<8.5/8.5–11/>11 × 10^9^ l^−1^)	75/23/7	37/33/22	10/11/15	<0.001
Neutrophil count (<7.5/>7.5 × 10^9^ l^−1^)	96/9	65/27	17/19	<0.001
Lymphocyte count (>3.0/1.0–3.0/<1.0 × 10^9^ l^−1^)	3/97/5	6/74/12	0/26/10	0.002
Monocyte count (<0.9/>0.9 × 10^9^ l^−1^)	95/10	74/18	24/12	0.001
Platelet count (<400/>400 × 10^3^ l^−1^)	93/11	62/30	17/18	<0.001
Neutrophil/lymphocyte ratio (<5/>5)	96/9	61/31	15/21	<0.001

TNM=tumour, node, metastases; mGPS=modified Glasgow Prognostic Score.

a Individual deprivation categories were used in the statistical analysis.
